# In-Situ Synthesized Si@C Materials for the Lithium Ion Battery: A Mini Review

**DOI:** 10.3390/nano9030432

**Published:** 2019-03-14

**Authors:** Wenmao Tu, Ziyu Bai, Zhao Deng, Haining Zhang, Haolin Tang

**Affiliations:** State Key Laboratory of Advanced Technology for Materials Synthesis and Processing, Wuhan University of Technology, Wuhan 430070, China; tuwm@whut.edu.cn (W.T.); 18971142560@163.com (Z.B.); dengzhao@whut.edu.cn (Z.D.); Haining.zhang@whut.edu.cn (H.Z.)

**Keywords:** lithium ion battery, Si@C compound material, in-situ synthesis

## Abstract

As an important component, the anode determines the property and development of lithium ion batteries. The synthetic method and the structure design of the negative electrode materials play decisive roles in improving the property of the thus-assembled batteries. Si@C compound materials have been widely used based on their excellent lithium ion intercalation capacity and cyclic stability, in which the in-situ synthetic method can make full use of the structural advantages of the monomer itself, thus improving the electrochemical performance of the anode material. In this paper, the different preparation technologies and composite structures of Si@C compound materials by in-situ synthesis are introduced. The research progress of Si@C compound materials by in-situ synthesis is reviewed, and the prospect of future development of Si@C compound materials has been tentatively commented.

## 1. Introduction

The lithium ion battery is one of the ideal green rechargeable energy conversion devices. Its principle is based on the lithium ion intercalation and deintercalation between a carbon negative electrode and a compound positive electrode. Lithium ion batteries have been widely used in portable electronic products because of their advantages, including large energy density, small self-discharge, high output voltage, and good security [1,2]. The commercially available anode materials for lithium ion batteries are mainly graphite and other carbonaceous materials. However, the poor rate performance indicates that it is difficult to meet the demand of downstream products of lithium-ion batteries. In the field of consumer electronics, the energy density of the battery needs to be improved. The Si@C compound material, which is a new kind of high-capacity negative materials, will become the development trend of lithium ion batteries.

The main advantages of silicon-based materials are depicted as follows. (1) The excellent capacity [3]; (2) the relatively stable amorphous microstructure after the first lithium intercalation; (3) difficulty in reunion of materials during the process of lithium ion deintercalation. In addition, it is also not easy to generate the lithium dendrite, because of the higher discharge platform compared to carbon-based materials, and there is no co-embedding of electrolytes. The main disadvantage is the serious volume effect during the process of high lithium ion deintercalation, resulting in the collapse of the electrode materials and the exfoliation of active substances, characterizing a rapid capacity attenuation [4]. Based on the advantages and disadvantages of silicon materials and carbon materials, it is a good choice to synthesize Si@C compound materials, with an optimized design of structure, as the negative material [5]. In such a system, silicon is used as an active substance to provide lithium storage capacity [6]; carbon is used as a dispersed matrix to buffer the volume effect of silicon particles in the process of charging–discharging, in order to maintain the electrical contact within the electrode and the integrity of the electrode structure [7]. The preparation of Si@C anode materials by using the in-situ synthesis method can make full use of the structural advantages of the monomer, and creates a good fusion of the advantages of their respective properties, thus improving the performance of the composites. 

Numerous lithium-ion Si@C anode materials have been designed to buffer the volume expansion of silicon and to optimize the lithium intercalation performance of silicon, by controlling synthetic methods and designing different structural models. For example, Si@C composites are prepared by efficient mixing using high-energy ball-milling, or by adding the eff to link the silicon-containing materials and carbon-containing materials, so as to achieve different structural designs for improvement in electrochemical properties. Aiming at in-situ synthesis, this method is developed on the basis of enhancing the mechanical and physical active surfactant properties of inorganic materials. Based on the advantages of in-situ synthesis, it has been extensively applied to organic synthesis and adsorption processes in environmental engineering, but it is seldom used in energy storage. By analyzing the properties of the required composites, suitable in-situ monomers are selected to retain the performance advantages of the monomers in the system, and the structural advantages of the monomers can also be designed. For Si@C anode materials of lithium-ion batteries, high performance anode materials can be prepared by in-situ electrochemical synthesis using alloying products during charging and discharging, and solid-phase in-situ synthesis can also be carried out on the basis of raw material monomers. The in-situ synthesis method based on environmental protection adsorbent materials is applied to prepare the components of lithium storage devices, which is a novel development direction of negative electrode composite for lithium ion batteries. 

## 2. In-Situ Synthesis of Si@C Compound Materials

Generally, the synthetic process which is based on a hybrid precursor comprising both silicon components and organic components, and involves simultaneous formation of active electrode materials and carbonaceous protective coating is referred to an “in-situ synthesis”. In-situ synthesis is a recently developed technology for preparing compound materials. The basic principle relies on the occurred chemical reaction between different elements or compounds under a certain condition and the generated secondary phases in the substrate phase to improve the performance of single substrate. In such a compound system, reinforcement spontaneously grows in the substrate phase and the interfacial bonding strength can be improved. For in-situ synthesis, if there are organic compounds in the synthetic monomers, organic–inorganic polymers are often formed. Inorganic molecules can be grafted on the organic monomers to form a homogeneous composite of an encapsulated type, rather than a simple physical compound formed by mechanical mixing. If the monomers used for synthesis are inorganic, the co-embedding of monomers can be realized. The bonding strength is obviously greater than the physical force of mechanical mixing, so that the properties of the composite will fully combine the advantages of monomer properties. Compared with ex-situ synthesis, the structure of the composites synthesized is more uniform, and the design of specific structures can be achieved by the selection of monomers. 

### 2.1. In-Situ Electrochemical Synthesis 

In the process of alloying and dealloying of lithium ion with Si, silicon-based materials will cause severe structural damage due to its huge volume effect. Thus, the specific capacity is poor owing to the loss of electrical contact between the cracked and isolated Si particles [8,9]. However, based on the formation of a high lithium intercalation intermetallic compound and the appearance of amorphous phase in the material system, the silicon-based materials have high theoretical specific capacity. Therefore, how to reduce the shortcomings of silicon-based anode materials, reasonably, and fully tap their theoretical specific capacity will be the future research focus for silicon-based anode materials. To solve the problem of structural instability of silicon-based anodes, the Si@C composite by in-situ electrochemical synthesis has been developed. The in-situ method can generate the active–inactive product in which the silicon is evenly dispersed in the carbon matrix, to improve the mechanical properties and the electronic conductivity of materials [10,11,12]. This inactive or relatively active matrix exhibits good mechanical strength to buffer the stress and strain generated by the charge and discharge process of the active silicon phase. In addition, the substrate with electrochemical active phase must have high conductivity and lithium ion mobility to make the material possess a high rate performance.

At the initial stage of the charge and discharge process, the electrochemical cycle is limited in the Li-Si intermetallic compound, rather than in the silicon particles, which would help to alleviate the volume effect during the alloying process. Moni et al. [13] reported the generation of lithialized Si@C compound material by in-situ electrochemical synthesis using high energy mechanical milling (HEMM). The theoretical specific capacity of the Li-Si@C composite based on different Li-Si alloy compositions is shown in Table 1. In this report, multiple contrasting samples of Li-Si@C were prepared by mixing the Si@C composite prepared by HEMM and PAN (polyaniline) with 42 wt.% Gr, 28 wt.% Si, and 30 wt.% PAN under various electrochemical reaction conditions and different components. The materials were obtained at ~0.6 V, ~0.5 V, and ~0.4 V, with the Li-Si alloy dispersed in graphite matrix. Under constantly cycling at stable potential, the composite exhibited better cyclability in comparison to the pure Si@C composite. The energy density was improved because of the higher discharge potential for the full cell. The Li-Si@C compound material with 64 at.%C, 21.6 at.% Li, and 14.4 at.% Si could have a stable cycle in the potential window of 0.02–0.5 V, which is similar to the composite with Li-40 at.% Si dispersed in the carbon matrix, and the composite obtains a high energy density in the full cell configuration with excellent stability (~0.13% loss per cycle and capacity of 700 mAhg^−1^).

### 2.2. In-Situ Solid-State Synthesis

Yushin et al. reported on the coated nano silicon@graphene composite particle that is prepared by the method of a layered, bottom-up assembly, through continuous silicon and carbon chemical vapor deposition (CVD) processes, started from multilayer graphene gel [14,15]. After accurately creating and adjusting nano-scale pores in self-assembled particles, the three-dimensional porous particle structure, consisting of a curved two-dimensional layer, can be used as a buffer to adapt to the volume expansion of silicon and provide irregular channels for rapid acquisition of lithium ion transport, thus improving the reversible capacity, columbic efficiency, and cyclability. Although the CVD process using high purity propylene and highly flammable silane have a high cost, which may impede its commercial applications, this bottom-up strategy can be used to prepare hierarchical complex substances that have a long cycle life. Therefore, the direct synthesis of graphene-based composites by in-situ growth of graphene is considered as a new important strategy. 

**Figure 1:**
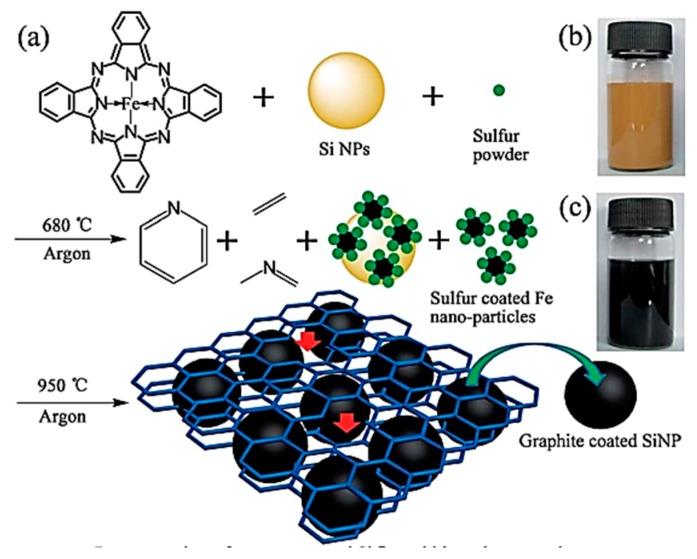
(**a**) Schematic diagram of the formation of the N-doped porous graphene frame-supported Si@graphite carbon granules; (**b**,**c**) images of SiNPs and as-prepared granules dispersed in ethanol solution.

Zhang et al. [16] reported the synthesis of silicon@graphitic carbon via a one-step in-situ solid-state reaction. The schematic diagram of the formation of the N-doped porous graphene frame-supported Si@graphite carbon and the cycling performance of SiNPs, Graphene, and N-doped graphene frame-supported Si@graphite carbon (NGSi@G) granules are shown in Figure 1. In this system, the in-situ-formed nitrogen-doped graphene wrapped the SiNPs through thermal decomposition and re-carbonization of FePC, forming a cross-linked graphene skeleton network, and SiNP was encapsulated by a thin graphite carbon layer. The self-assembly process occurred based on the strong interconnections between the graphene, graphite carbon, and SiNPs, resulting in the formation of nanoparticles with tunable nano-porosities and irregular channels. The self-assembled particles with good conductivity can effectively adapt to the volume change of SiNPs during the reduplicate charge–discharge process, thus achieving high capacity, long cycle life, high coulomb efficiency, and excellent rate capacity. The results show that the graphene frame-supported silicon@graphitic carbon granules have high reversible capacities of 1345 and 1065 mAhg^−1^ after 100 and 200 cycles, respectively, corresponding to capacity retentions of 91% and 72%, respectively. A high reversible capacity of 1042 mAhg^−1^ at 28 Ag^−1^ (10C) can also be achieved, revealing a superb rate capability.

### 2.3. In-Situ Carbothermal Reduction

Carbon thermal in-situ synthesis has its unique advantages: (1) The decomposition products of high molecular polymers have higher reducibility than solid carbon, which can reduce the synthesis temperature and shorten the reaction time; (2) the derived carbon from polymer are atomically dispersed in the reaction system, leading to the homogeneous coating of synthetic products; (3) in-situ-coated carbon films can reduce the growth rate of LiFePO_4_. By using in-situ synthesis depending on chemical reaction, nanoscale silicon can be generated and it will be well-dispersed in the buffer matrix to form the composite. The prepared composite can suppress the absolute volume change and the cyclic stability of the material system can be improved accordingly [12,17,18]. Zhang et al. used the carbon thermal in-situ reduction method to prepare nano silicon (less than 50 nm) that was dispersed in a carbon matrix [19]. Under inert atmosphere, the Si@C composite was prepared by milling silicon oxide particles and sucrose, and sintering in a certain temperature. The composite with 20% Si, 80% C still maintained a high reversible capacity after 100 cycles, with an average attenuation rate of only 0.27%. The improvement in the cyclic performance of the Si@C compound material is mainly attributed to the effective buffer of the volume effect and the improved conductivity of graphite materials.

Rice husk is a natural material in which silicon and carbon sources coexist. The silicon dioxide is mostly concentrated on the upper and lower epidermis to form a compact silica shell, and a small amount of silica is distributed in its internal carbon material. The carbon materials, represented by lignin and cellulose, are mainly distributed in the internal vascular bundle [20]. Wang et al. carried out the pyrolysis of rice husk, graphitization at 1000 °C, and removed silicon dioxide to obtain rice hull-based carbon fibers. The material showed good capacity retention and rate capability [21]. Bao et al. obtained porous silicon from rice husk by use of the magnesium heat reduction method; the specific capacity could still reach 1220 mAhg^−1^ after 100 cycles at the current density of 1 Ag^−1^ and it also had a good rate performance [22]. Based on the preparation of rice husk silicon by magnesium heat reduction, Jung et al. prepared the Si@C compound material with a coated layer structure using poly(dopamine) as precursor. This material exhibits good electrochemical performance. The specific capacity of the first 200 cycles is above 1500 mAhg^−1^, and the rate capacity is good [23]. Wang et al. used alkali boiled in-situ carbothermal reduction to synthesize the Si@C compound anode material. In this method, the rice husk is pretreated with sodium hydroxide. After carbonization and magnesium heat reduction, the Si@C compound anode material was obtained. The composite has excellent rate capacity [24]. Si@C compound anode material can be also prepared by in-situ synthesis of different ash template carbons. 

## 3. The Structure and Electrochemical Properties of Si@C Compound Materials

For the coated composite, the surface carbon layer is mainly amorphous carbon [14], and for the embedded composite, the surface carbon layer can be graphite [25,26] or graphene. The three-component composite structure between silicon and carbon is also a hot topic at present. The synthetic method and electrochemical performance comparison of Si@C-based anode materials in the lithium ion battery are shown in Table 2.

### 3.1. Coated Composite

In the coated silicon–carbon composites, the high silicon content contributes to the increase in the lithium storage capacity, and the carbon layer coated on the surface of silicon can effectively alleviate the volume effect of silicon and enhance the electronic conductivity. For this system, a stable solid electrolyte membrane (SEI) can be produced to stabilize the interface between the electrode material and the electrolyte [27]. In order to solve the possible fracture of the carbon layer caused by intense silicon stress, research works to optimize the microstructure of the carbon layer and to prepare the nanofiber composite have been published [28].

**Figure 2:**
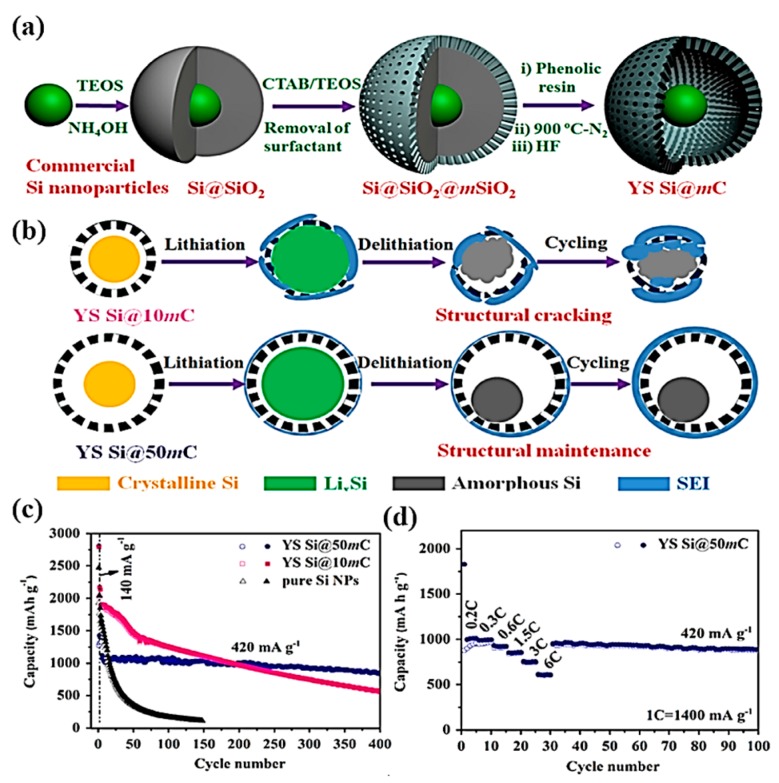
(**a**) Schematic illustration of the preparation of YS (yolk-shell) Si@mC; (**b**) schematic illustration of the lithiation–delithiation process of YS Si@10mC and YS Si@50mC; (**c**) cycling performance of pure Si NPs (nanoparticles), YS Si@10mC and YS Si@50mC; (**d**) rate performance of YS Si@50mC.

Gao et al. used in-situ polymerization and pyrolysis to prepare a Si@C compound material that had a core-shell structure, and its surface coating layer had a complete microporous structure. The negative electrode still maintained the reversible capacity of 1200 mAhg^−1^ after 40 cycles [29]. Cui et al. used the CVD method to deposit silicon on the surface of carbon fiber to prepare the nanowire Si@C compound material with a core-shell structure. After 30 cycles at the ratio of 0.2 C, the lithium storage capacity up to 2000 mAhg^−1^ was maintained [28]. Wang et al. [30] prepared a nano-silicon@C composite with a core-shell structure using a ball-milling and carbonization approach. In contrast to micro-silicon, the system with nano-silicon had a higher specific capacity. As a coating layer, carbon can make use of the buffering effect to improve the capacity retention. After 100 cycles, the specific capacity can be maintained at 1060 mAhg^−1^. It is well known that the existence of a core-shell structure can effectively solve the volume effect of silicon and improve the electrochemical performance. Zhou et al [31] synthesized Si/graphite@N-doped carbon with a core-shell structure and doping system using a two-step synthesis, including liquid mixing and spray-drying, to prepare silicon–graphite particles and oxidative self-polymerization of dopamine to compare coating layer. The composite exhibited a high specific capacity of 611.3 mAhg^−1^ after 100 cycles. As a development of the solid Si@C core-shell structure, the introduction of additional internal void spaces into the core-shell structure to form a yolk-shell structure can make particles contact better. The voids in this structure can buffer the volume expansion of silicon and allow the core to shrink without pulverization, which is beneficial to form stable SEI and to hold the integrity of the electrode. Yang et al [32] used a template-based and nanocasting method to synthesize a yolk-shell silicon-mesoporous carbon material. The schematic illustration and electrochemical tests are shown in Figure 2. In this structure, single silicon nanoparticles are encapsulated in an open and accessible mesoporous carbon layer rather than a solid carbon layer. In addition to providing enough space for volume expansion, the porosities of carbon shells allow rapid transport of lithium ions between electrolytes and silicon egg yolks. It was presented that the specific capacity was maintained at 1000 mAhg^−1^ after 400 cycles and the superior rate capability of 62.3% capacity retention at a current density of 8.4 Ag^−1^ was realized.

### 3.2. Embedded Composite

**Figure 3:**
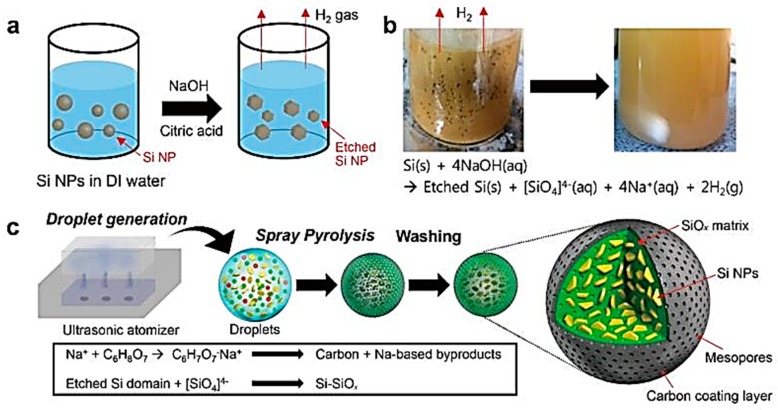
The synthesis illustration of the Si-SiOx@C compound material. (**a**) Schematic illustration of the preparation of the precursor solution. (**b**) Photographs of the precursor solution before and after the denoted reaction. (**c**) Graphical presentation for the synthesis of the Si−SiOx−C composite from the spray pyrolysis through a final washing step. Reprinted (adapted) with permission from [35], copyright 2017 American Chemical Society.

Because of the low silicon content of the embedded Si@C composite, its reversible capacity is relatively low; however, the high carbon content results in high stability [33]. The embedded composite refers to the embedding of silicon particles into the carbon matrix to form two particles, and the structure stability of the composite and the electrochemical activity of the electrode can be possibly improved depending on the conductive carbon medium. Wang et al. fabricated the embedded Si@C composite using a two-step CVD process, with which carbon nanotubes are grown, followed by the silica growth on carbon nanotubes. Through the physical force, silicon will embed into the carbon nanotube layered structure. The reversible capacity of the anode material was up to 2000 mAhg^−1^, and after 40 cycles the capacity was basically unattenuated [34]. Lee et al [35] reported a Si-SiOx@C compound material with an embedded structure and a surface carbon layer, as illustrated in Figure 3. In the system, SiO_4_^4−^ was transformed into a SiOx matrix by spray pyrolysis machine and citric acid was decomposed into a carbon layer. Therefore, the material presented high electrochemical performance (a reversible capacity of 1561 mAhg^−1^ at a rate of 0.06 C and, after 100 cycles, a capacity retention of 87.9% at a rate of 1 C). In the composite of Si–graphite, the cyclability is limited owing to the loose connections between flaked graphite particles and the poor interface adhesion among graphite and silicon. By introduction of another internal organic carbon source into the Si–graphite composite, the strong interfacial bonding can be formed and silicon can be evenly distributed into graphite. Datta et al. prepared a Si–graphite–PAN-C composite by the thermal decomposition of polyacrylonitrile (PAN)-based amorphous carbon (PAN-C), where the active Si phase was distributed in the graphite matrix. The introduced PAN acted as a diffusion barrier to inhibit the interfacial diffusion reaction between graphite and Si, so graphite retained its desired structure. This complete graphite was cut into ductile carbon matrix together with PAN-C to reduce the volume change of Si and suppress the irreversible loss of the anode. The composite exhibited a reversible capacity of 660 mAhg^−1^ with almost no decay up to 30 cycles at a constant current of 160 mAg^−1^.

### 3.3. Doped Composite

The Silicon–amorphous carbon–graphite three elements Si@C composite system is mainly prepared by ball-milling and high-temperature pyrolysis. In this system, the chemical properties of the material can be improved by modifying the porous structure of silicon [36]. The existence of silicon contributes to the increase of capacity, graphite contributes to the improvement of the dispersion of silicon particles [37], and the amorphous carbon plays the role of the binder. In this composite, the type of carbon is changeable. The mesoporous carbon@ carbon nanotubes@ amorphous carbon materials are prepared using magnesium thermal reduction and CVD method. After 400 cycles, the reversible capacity remains 710 mAhg^−1^. Wang et al. [38] reported Si@flake-graphite–amorphous-carbon by using ball-milling and spray drying. In this system, silicon and graphite were coupled by a PVP (polyvinyl pyrrolidone) binder, which was also used as a carbon source to form the coated layer. After high temperature treatment, a porous spherical shape was obtained. At a current density of 5 Ag^−1^, the capacity of 200 mAhg^−1^ was maintained. After 300 cycles, the gravimetric capacity of up to 400 mAhg^−1^ was obtained. Wu et al. [39] used electrostatic spray deposition and heat treatment to synthesize a Si–graphene–porous carbon composite that had a layer-by-layer porous carbon framework to suppress the volume effect of silicon and a flexible graphene layer to facilitate electron transport and maintain integrity of the system. The material displayed a reversible capacity of 1020 mAhg^−1^ after 100 cycles at a current density of 200 mAg^−1^. Agyeman et al. [40] synthesized the sandwich structure of Si@C-rGO by using stirring and vacuum filtration. Owing to the sandwich structure, with strong covalent and hydrogen bonding, the materials presented an excellent rate capacity of 767 mAhg^−1^ at a current density of 3 Ag^−1^, high gravimetric capacity of 1001 mAhg^−1^ at 300 mAg^−1^, and a great cyclic stability. 

## 4. Conclusions and Prospect

The cycle stability and the reversible capacity retention rate are two important properties in the development of lithium ion batteries. It also restricts the commercialization of silicon carbon composites. By exploring the essential factors that affect its performance, the structure of composite materials is optimized to solve the problem of performance. In this paper, the recent research progress of in-situ synthesis of silicon carbon composites is described. The following suggestions are put forward for the future development of anode materials for lithium ion batteries: (1) Seeking a monomer with such kind of advantages, including the inorganic core and the organic shell of siliceous material; (2) optimizing the microstructure of composite material by a more effective preparation method; (3) combining the nano size with the mesoporous structure by the ternary system; (4) the combination of multiple forms achieves multiple performance capability. 

In the system of in-situ electrochemical synthesis of Si@C anode materials, a new concept where the lithium-intercalated silicon alloy is uniformly dispersed in the carbon matrix materials has been proposed, which can make its capacity retention rate steady in a low voltage window without affecting the total energy density. By dispersing the lithium-intercalated alloys in different states into the matrix, and by preparing the anode materials with different contents of component, the electrochemical properties of the materials under different voltage windows can be investigated. Furthermore, it can also explain the lithium-intercalation mechanism of the anode materials in the process of charging–discharging. This method provides a new possibility for the preparation of anode materials and on the application and production with further development. In the system of in-situ solid-state synthesis, through the in-situ growth of graphene, the one-step solid-state synthesis of Si@C composites can be achieved. The composite takes full advantage of the excellent properties of graphene, which has high conductivity and can effectively regulate the charge–discharge cycle. This self-assembly method is simple, safe, and low-cost. It is helpful to synthesize graphene-based composites with different functions that can be applied to the lithium-ion batteries, supercapacitors, and fuel cells. In the system of in-situ carbothermal synthesis of Si@C anode materials, the raw materials are natural rice husk materials and the composites are prepared by a typical large-scale carbothermal reduction treatment. It is an environmentally friendly preparation method, which has the potential of large-scale production. Looking at the whole lithium-ion battery anode material industry, Si@C anode is still in the infancy stage, and its commercial application still needs further development. Nevertheless, in-situ synthesis provides a new direction for the preparation of Si@C anode materials. In the process of seeking high performance, the mechanism of internal electrochemical changes can also be studied, providing possibility for the improvement of the performance and industrialization.

## Figures and Tables

**Table 1:** The theoretical specific capacity with Li-Si–C composite based on different Li-Si alloy compositions.

Alloys	Composition	Phases	Specific Capacity (mAhg^−1^)	Capacity Loss per Cycle (%)	Charge Potential (V)
Si/C	82.6 at.% C, 18.4 at.% Si	Si + C	1000	0.34	1.2
Li-Si/C(0.6 V)	69 at.% C, 15.5 at.% Si, 15.5 at.% Li	Li-Si + C	800	0.21	0.6
Li-Si/C(0.5 V)	64 at.% C, 21.6 at.% Si, 14.4 at.% Li	Li-Si + Li7Si3 + C	700	0.13	0.5
Li-Si/C(0.4 V)	57.1 at.% C, 30 at.% Si, 12.9 at.% Li	Li7Si3 + C	440	3.6	0.4
C	-	C	320	0%	1.2
Si	-	Si	4000	-	1.2

**Table 2:** Synthesis method and electrochemical performance comparison of Si@C-based anode materials in the lithium ion battery.

Anodes	Specific Capacity (mAhg^−1^)	Cycling Stability (mAhg^−1^)	Rate Capacity (mAhg^−1^)	Structure	Method	Ref
Li-Si/C	1019	821 after 30 cycles at 160 mAg^−1^	-	Li-Si alloys in carbon matrix	In-situ electrochemical	[20]
Si@graphitic	1479	1065 after 200 cycles at 280 mAg^−1^	1042 at 28 Ag^−1^	Nanosilicon-coated graphene granule	In-situ solid-state	[23]
Si@amorphous C	1291	650 after 100 cycles at 200 mAhg^−1^	-	Nanosilicon amorphous carbon core-shell	In-situ carbothermal reduction	[27]
LRP-Si@C	2110	1633 after 70 cycles at 0.5 Ag^−1^	580 at 8 Ag^−1^	Lotus root-like porous	Magnesiothermic reduction and CVD	[28]
Microporous Si@C	1887	1210 after 40 cycles at 0.5 C	-	Nano core-shell	In-situ polymerization	[29]
Si@SiOx@C	1980	1450 after 100 cycles at 0.1 Ag^−1^	1230 after 100 cycles (500 mAg^−1^)	Double-walled core-shell	Ball-milling and carbonization	[30]
Si/C	741.2	611.3 after 100 cycles at 0.3 Ag^−1^	480.3 at 4 Ag^−1^	Si/graphit@N-doped carbon core-shell	Spray-dying and carbonization	[31]
